# Association between dyslipidemia and diagnoses related to sensorineural hearing loss: a retrospective case–control study

**DOI:** 10.3389/fneur.2026.1979454

**Published:** 2026-10-28

**Authors:** Meng Yang, Qiang Liu, Xingke Tong, Lei Liu, Fuqiong Zhang, Yongdong Sun

**Affiliations:** 1 School of Integrated Traditional Chinese and Western Medicine, Southwest Medical University, Luzhou, Sichuan, China; 2 Department of Otorhinolaryngology, Affiliated Traditional Chinese Medicine Hospital of Southwest Medical University, Luzhou, Sichuan, China

**Keywords:** case–control study, dyslipidemia, lipid metabolism, retrospective study, sensorineural hearing loss

## Abstract

**Objective:**

To examine the association between dyslipidemia and sensorineural hearing loss–related diagnoses among otologic inpatients.

**Methods:**

This single-center retrospective case–control study included 580 otologic inpatients who underwent lipid and metabolic testing at the Department of Otorhinolaryngology, Affiliated Traditional Chinese Medicine Hospital of Southwest Medical University, from January 1, 2022 to December 31, 2024. Of these, 284 patients had sensorineural hearing loss–related diagnoses and 296 served as controls. Demographic characteristics, body mass index, blood pressure, glucose metabolism, lipid profiles, and renal function–related indicators were collected. Associations between lipid parameters and the outcome were assessed using univariate and multivariable logistic regression models. Robustness was further evaluated through sensitivity analyses with a narrower outcome definition, categorical lipid variables, restricted cubic spline models, quartile-based trend analyses, and subgroup and interaction analyses.

**Results:**

The case group had higher triglyceride, low-density lipoprotein cholesterol, body mass index, and uric acid levels, and lower high-density lipoprotein cholesterol levels than the control group, with statistically significant differences. After adjustment for age, sex, and body mass index, higher triglyceride levels were associated with increased odds of the outcome (OR = 1.195, 95% CI: 1.012–1.411, *p* = 0.036), as were higher low-density lipoprotein cholesterol levels (OR = 1.268, 95% CI: 1.030–1.562, *p* = 0.025). In contrast, higher high-density lipoprotein cholesterol levels were associated with lower odds of the outcome (OR = 0.484, 95% CI: 0.290–0.807, *p* = 0.005). After further adjustment for available metabolic covariates, triglycerides (OR = 1.202, 95% CI: 1.015–1.423, *p* = 0.033), low-density lipoprotein cholesterol (OR = 1.284, 95% CI: 1.039–1.588, *p* = 0.021), and high-density lipoprotein cholesterol (OR = 0.501, 95% CI: 0.299–0.842, *p* = 0.009) remained significantly associated with diagnoses related to sensorineural hearing loss. These associations remained generally consistent in sensitivity analyses restricted to sudden deafness or sudden hearing loss and in models using categorical lipid variables. Restricted cubic spline and quartile analyses suggested dose-related associations, with higher triglyceride and lower high-density lipoprotein cholesterol levels linked to increased odds of the outcome.

**Conclusion:**

Among otologic inpatients, higher triglyceride and low-density lipoprotein cholesterol levels, together with lower high-density lipoprotein cholesterol levels, were associated with sensorineural hearing loss–related diagnoses. These findings were generally consistent across supplementary analyses, suggesting that dyslipidemia may represent a metabolic feature in this patient population.

## 1 Introduction

Sensorineural hearing loss (SNHL) is a common auditory impairment encountered in otorhinolaryngology. In some patients, substantial hearing deterioration may occur within a short period, highlighting the clinical value of early identification and timely intervention (1). Depending on the mode of onset, the range of affected frequencies, and the wording of clinical diagnoses, this condition may be recorded clinically as sudden deafness, sudden hearing loss, or sensorineural hearing loss. Unlike conductive hearing loss, SNHL primarily involves cochlear hair cells, the auditory nerve, or central auditory pathways and has a distinct pathological and risk-factor profile (2, 3). Its etiology is multifactorial, with inner-ear microcirculatory disturbance, oxidative stress, inflammation, immune abnormalities, and metabolic dysregulation implicated in its development (3–5). Dyslipidemia may affect the inner-ear microenvironment through vascular, inflammatory, and oxidative mechanisms (4–6). In contrast, conductive hearing loss primarily arises from external- or middle-ear pathology. Mixed hearing loss contains both conductive and sensorineural components; cases with a documented sensorineural component were therefore retained within the broader SNHL-related diagnostic spectrum (7).

Current findings regarding the relationship between lipid parameters and SNHL remain inconsistent. Existing studies differ with respect to population source, case definition, control selection, covariate adjustment, and the availability of audiological data (2, 8, 9). Most previous studies have used healthy or general-population controls. In contrast, the present study used contemporaneous otologic inpatients as controls, which better reflects the clinical setting in which metabolic testing is routinely performed, although such controls are inherently more heterogeneous (6, 9, 10).

Based on these considerations, the present study used real-world data from inpatients in the Department of Otorhinolaryngology, Affiliated Traditional Chinese Medicine Hospital of Southwest Medical University, with SNHL-related diagnoses defined as the primary outcome. We systematically evaluated the associations of lipid parameters, including triglycerides (TG), total cholesterol (TC), high-density lipoprotein cholesterol (HDL-C), and low-density lipoprotein cholesterol (LDL-C), with the outcome. The robustness and consistency of the findings were further assessed using multivariable models, sensitivity analyses, subgroup and interaction analyses, restricted cubic spline (RCS) analysis, and quartile-based trend analysis.

## 2 Materials and methods

### 2.1 Study design and data source

This was a single-center, retrospective, case–control study. The study population consisted of otologic inpatients admitted to the Department of Otorhinolaryngology, Affiliated Traditional Chinese Medicine Hospital of Southwest Medical University, between January 1, 2022 and December 31, 2024. All included patients underwent testing for lipid profiles and related metabolic indicators during hospitalization. Lipid profiles and blood glucose were obtained from the first fasting blood sample during hospitalization. For patients who were not fasting at admission, blood sampling was performed on the following morning after an overnight fast. This study was a secondary analysis based on existing electronic medical records and laboratory data, and no additional diagnostic or therapeutic interventions were performed.

### 2.2 Study population

Patients with sudden deafness, sudden hearing loss, sensorineural hearing loss, or mixed hearing loss with a sensorineural component recorded among the main discharge diagnoses during the index hospitalization were classified as cases; historical diagnoses were not used for grouping. SNHL-related diagnoses were established by otorhinolaryngologists based on medical history, otologic examination, and routine audiological evaluation. The control group consisted of other eligible otologic inpatients without these SNHL-related discharge diagnoses. Because diagnostic terms such as “hearing decline” and “reduced hearing” lack sufficient specificity and may be related to external or middle ear lesions, otologic trauma, tinnitus-related complaints, or other non-sensorineural factors, they were not used as inclusion criteria for the case group. This approach was intended to reduce outcome misclassification and improve the specificity of the case definition. The main diagnoses in the control group included external auditory canal diseases, middle-ear inflammatory diseases, tinnitus, and vestibular disorders.

The inclusion criteria were as follows:

1. admission to the otorhinolaryngology inpatient ward of the Affiliated Traditional Chinese Medicine Hospital of Southwest Medical University;
2. complete inpatient diagnostic records allowing determination of outcome grouping;
3. completion of lipid profile and related metabolic testing;
4. age ≥18 years.

The exclusion criteria were as follows:

1. severely incomplete diagnostic information or inability to determine the outcome;
2. substantial missing data for the main exposure variables;
3. hearing loss clearly caused by special factors such as trauma, tumor, surgery, or drug toxicity and not meeting the study outcome definition;
4. duplicate hospitalization records that could not be merged or adjudicated.

### 2.3 Variable definitions

The primary outcome was the presence of an SNHL-related discharge diagnosis as defined above. The main exposure variables were lipid metabolic indicators, including triglycerides (TG), total cholesterol (TC), high-density lipoprotein cholesterol (HDL-C), and low-density lipoprotein cholesterol (LDL-C). Considering the biological correlations of TC with TG, HDL-C, and LDL-C, the main multivariable models included TG, HDL-C, and LDL-C as the core lipid exposures, whereas TC was used primarily for descriptive and univariate analyses.

Collected covariates included age, sex, body mass index (BMI), systolic blood pressure (SBP), diastolic blood pressure (DBP), blood glucose (BG), creatinine (CRE), uric acid (UA), and urea. Age, BMI, blood pressure, lipid parameters, blood glucose, and renal function/uric acid metabolism indicators were included as continuous variables in the main analyses. In sensitivity analyses using categorical lipid variables, binary indicators such as elevated TG, elevated LDL-C, and reduced HDL-C were included according to the predefined classification variables in the dataset.

### 2.4 Statistical analysis

Continuous variables were presented as means ± standard deviations or medians with interquartile ranges, depending on their distribution. Categorical variables were expressed as counts and percentages. Between-group comparisons were performed using the independent-samples *t*-test, Mann–Whitney *U* test, or *χ*^2^ test, as appropriate.

Using diagnoses related to sensorineural hearing loss as the outcome variable, univariate logistic regression was first performed to examine crude associations, followed by prespecified multivariable logistic regression models. A basic demographic model was first constructed with adjustment for age, sex, and BMI. TG, HDL-C, and LDL-C were then added to the basic demographic model to form the lipid metabolism model. Finally, blood glucose, uric acid, and diastolic blood pressure were further included in the lipid metabolism model to obtain the extended metabolic model. Results were reported as odds ratios, 95% confidence intervals, and *p-*values.

Several supplementary analyses were performed to assess the robustness of the findings. The outcome was restricted to sudden deafness or sudden hearing loss, and lipid parameters were additionally analyzed using predefined clinical categories. Restricted cubic spline and quartile-based analyses were used to assess nonlinearity and dose–response patterns, while subgroup and interaction analyses were conducted according to sex, age, and BMI. An additional sensitivity analysis restricted the control group to patients with external- or middle-ear disorders. Given the retrospective design, no *a priori* sample-size calculation was performed, and all eligible patients during the predefined study period were included. A sensitivity power analysis was conducted to estimate the minimum detectable effect size.

### 2.5 Ethical approval and informed consent

This retrospective case–control study was approved by the Medical Ethics Committee of the Affiliated Traditional Chinese Medicine Hospital of Southwest Medical University (approval No. BY2026049). The requirement for informed consent was waived by the ethics committee because this study used de-identified retrospective clinical data extracted from electronic medical records. All procedures were conducted in accordance with the Declaration of Helsinki.

## 3 Results

### 3.1 Study population and baseline characteristics

A total of 580 patients were included, comprising 284 cases and 296 controls. Except for three missing values each for HDL-C and LDL-C, the main variables were complete. Compared with controls, cases had higher TG, LDL-C, BMI, and UA levels and lower HDL-C levels, whereas the remaining baseline variables did not differ significantly (Table 1). The diagnostic distributions are presented in Supplementary Table 4.

**Table 1:** Comparison of baseline and metabolic indicators between the control and case groups.

Variable	Control group	Case group	*p-*value
Male sex, *n* (%)	123/296 (41.6)	136/284 (47.9)	0.147
Age, years	53.000 [43.000, 61.250] (*n* = 296)	55.000 [46.750, 63.250] (*n* = 284)	0.103
BMI, kg/m^2^	23.854 [21.380, 25.788] (*n* = 296)	24.262 [22.143, 27.181] (*n* = 284)	0.024
TG, mmol/L	1.305 [0.890, 1.940] (*n* = 296)	1.605 [1.108, 2.390] (*n* = 284)	<0.001
TC, mmol/L	4.915 [4.348, 5.670] (*n* = 296)	5.075 [4.428, 5.820] (*n* = 284)	0.154
HDL-C, mmol/L	1.420 [1.170, 1.720] (*n* = 294)	1.280 [1.060, 1.595] (*n* = 283)	<0.001
LDL-C, mmol/L	2.880 [2.353, 3.465] (*n* = 294)	3.020 [2.480, 3.620] (*n* = 283)	0.017
BG, mmol/L	5.505 [4.920, 6.600] (*n* = 296)	5.700 [5.000, 6.900] (*n* = 284)	0.150
UA, μmol/L	303.000 [253.750, 372.250] (*n* = 296)	321.500 [261.750, 408.000] (*n* = 284)	0.016
SBP, mmHg	122.000 [111.750, 133.250] (*n* = 296)	123.000 [113.000, 136.000] (*n* = 284)	0.664
DBP, mmHg	78.000 [71.000, 87.000] (*n* = 296)	78.000 [72.000, 86.000] (*n* = 284)	0.806
CRE, μmol/L	65.000 [56.000, 75.000] (*n* = 296)	67.000 [56.000, 79.000] (*n* = 284)	0.117
Urea, mmol/L	5.005 [4.170, 5.985] (*n* = 296)	4.945 [4.107, 6.095] (*n* = 284)	0.604

Data are presented as *n* (%) for categorical variables and median [*Q*1, *Q*3] for continuous variables. *p* < 0.05 was considered statistically significant.

### 3.2 Univariate logistic regression

The results of univariate logistic regression are shown in Table 2. TG, HDL-C, UA, LDL-C, and BMI were statistically associated with the outcome. Higher TG and LDL-C levels were positively associated with case-group diagnoses, whereas higher HDL-C levels were negatively associated with the outcome. Age, sex, TC, BG, blood pressure, CRE, and urea did not reach statistical significance in the univariate logistic regression models.

**Table 2:** Results of univariate logistic regression analysis.

Variable	OR (95%CI)		*p-*value
HDL-C	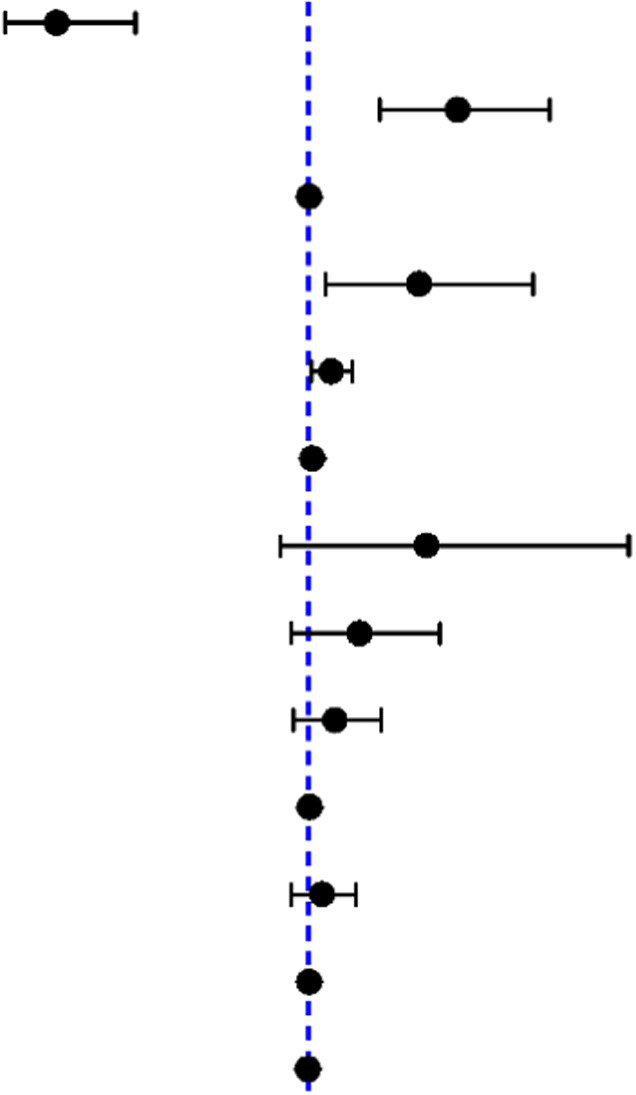	0.375 (0.247–0.570)	<0.001
TG		1.371 (1.177–1.598)	<0.001
UA		1.002 (1.001–1.004)	0.007
LDL-C		1.274 (1.043–1.557)	0.018
BMI		1.056 (1.007–1.108)	0.025
Age		1.009 (0.998–1.021)	0.118
Sex		1.292 (0.931–1.794)	0.125
TC		1.127 (0.957–1.326)	0.152
Urea		1.066 (0.963–1.180)	0.216
CRE		1.004 (0.996–1.011)	0.330
BG		1.033 (0.956–1.117)	0.407
SBP		1.001 (0.992–1.011)	0.790
DBP		0.999 (0.985–1.014)	0.908

OR > 1 indicates a positive association, whereas OR < 1 indicates a negative association. *p* < 0.05 was considered statistically significant.

### 3.3 Multivariable logistic regression analysis

Analyses were performed according to the three prespecified multivariable logistic regression models: the basic demographic model, the lipid metabolism model, and the extended metabolic model. Multivariable analyses were conducted using complete-case analysis. The results are shown in Table 3 and Figure 1. In the basic demographic model, age, sex, and BMI were not significantly associated with the study outcome (all *p* > 0.05). After further inclusion of TG, HDL-C, and LDL-C in the lipid metabolism model, TG (*p* = 0.036) and LDL-C (*p* = 0.025) were positively associated with the outcome, whereas HDL-C (*p* = 0.005) was negatively associated with the outcome; all three associations reached statistical significance. After further adjustment for BG, UA, and DBP in the extended metabolic model, the directions of association for these lipid parameters remained consistent, and TG (*p* = 0.033), LDL-C (*p* = 0.021), and HDL-C (*p* = 0.009) remained statistically significant. In contrast, age, sex, BMI, and other metabolic covariates showed no significant associations.

**Table 3:** Results of multivariable logistic regression models.

Model	Variable	OR (95%CI)	*p-*value
Basic demographic model	BMI	1.047 (0.997–1.100)	0.063
Basic demographic model	Age	1.009 (0.997–1.021)	0.129
Basic demographic model	Sex	1.264 (0.903–1.769)	0.173
Lipid metabolism model	HDL-C	0.484 (0.290–0.807)	0.005
Lipid metabolism model	LDL-C	1.268 (1.030–1.562)	0.025
Lipid metabolism model	TG	1.195 (1.012–1.411)	0.036
Lipid metabolism model	Age	1.005 (0.993–1.017)	0.429
Lipid metabolism model	BMI	1.005 (0.954–1.058)	0.863
Lipid metabolism model	Sex	1.030 (0.717–1.481)	0.871
Extended metabolic model	HDL-C	0.501 (0.299–0.842)	0.009
Extended metabolic model	LDL-C	1.284 (1.039–1.588)	0.021
Extended metabolic model	TG	1.202 (1.015–1.423)	0.033
Extended metabolic model	DBP	0.989 (0.973–1.005)	0.158
Extended metabolic model	Age	1.005 (0.992–1.017)	0.474
Extended metabolic model	UA	1.000 (0.998–1.003)	0.642
Extended metabolic model	BMI	1.011 (0.957–1.068)	0.690
Extended metabolic model	BG	1.011 (0.931–1.098)	0.789
Extended metabolic model	Sex	1.040 (0.702–1.539)	0.846

The basic demographic model included age, sex, and BMI. The lipid metabolism model further included TG, HDL-C, and LDL-C on the basis of the basic demographic model. The extended metabolic model further included BG, UA, and DBP. OR > 1 indicates a positive association, whereas OR < 1 indicates a negative association. *p* < 0.05 was considered statistically significant.

**Figure 1:**
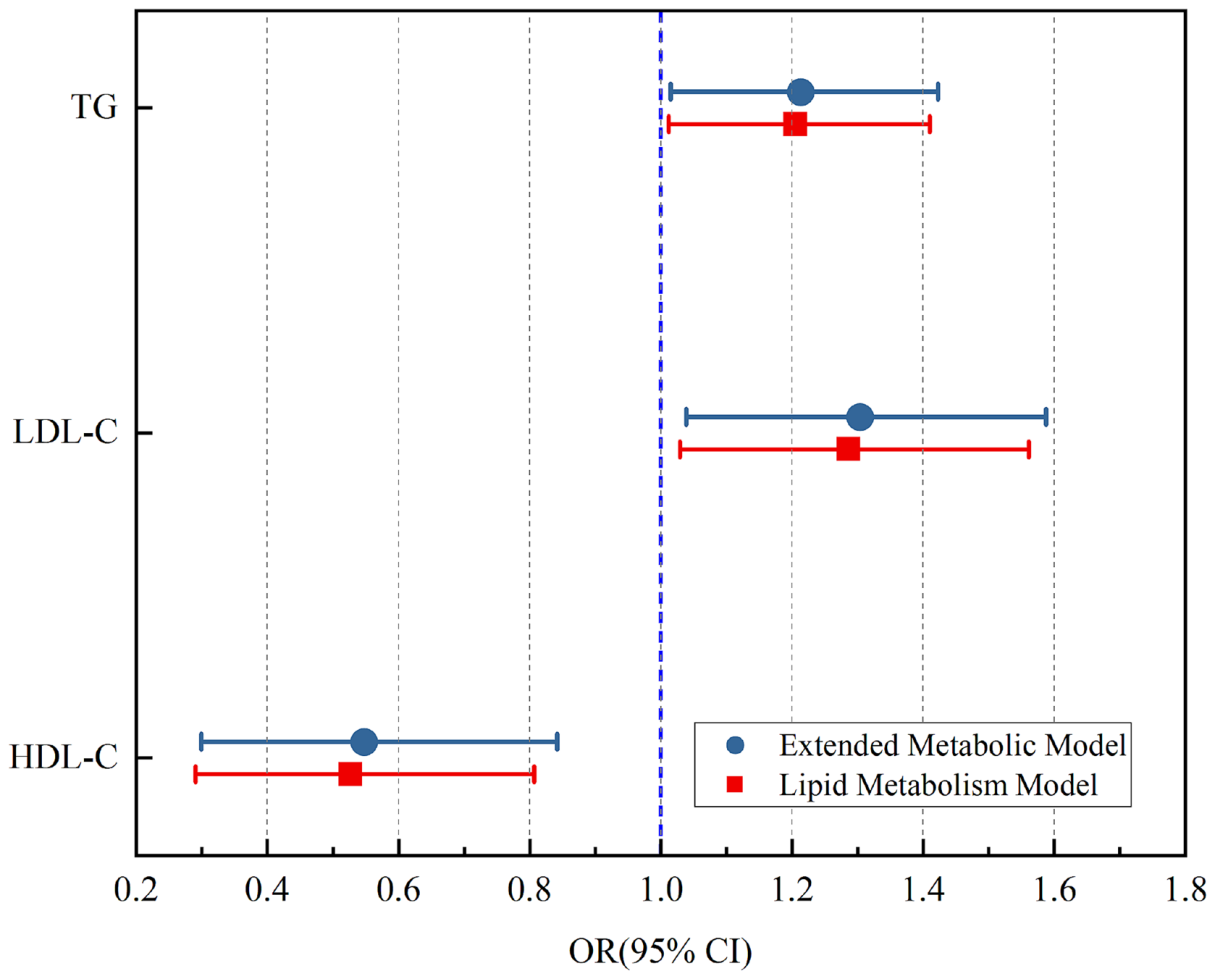
Forest plot of multivariable logistic regression for lipid parameters.

### 3.4 Sensitivity analyses

Several sensitivity analyses were conducted to evaluate the robustness of the main findings. In the first analysis, the case group was restricted to patients with sudden deafness or sudden hearing loss to assess whether the associations between the main lipid parameters and the outcome remained consistent under a narrower and more clinically specific outcome definition. This analysis included 527 patients, including 231 cases and 296 controls. Because of a small number of missing values for HDL-C or LDL-C, the lipid metabolism model was analyzed using complete cases, with 524 patients ultimately included.

When the outcome was restricted to sudden deafness or sudden hearing loss, TG and LDL-C remained positively associated with the outcome, whereas HDL-C retained an inverse association but did not reach statistical significance (Table 4). The directions of association remained similar after additional metabolic adjustment. Finally, using external- or middle-ear disorders as controls yielded similar directions of association for TG, LDL-C, and HDL-C, although confidence intervals widened and statistical significance was not retained (Supplementary Table 5).

**Table 4:** Sensitivity analysis under a narrower outcome definition.

Model	Variable	OR (95%CI)	*p-*value
Restricted-outcome lipid metabolism model	LDL-C	1.255 (1.012–1.557)	0.039
Restricted-outcome lipid metabolism model	TG	1.183 (1.001–1.400)	0.049
Restricted-outcome lipid metabolism model	HDL-C	0.621 (0.366–1.054)	0.077
Restricted-outcome extended metabolic model	LDL-C	1.266 (1.017–1.576)	0.035
Restricted-outcome extended metabolic model	TG	1.185 (1.001–1.404)	0.049
Restricted-outcome extended metabolic model	HDL-C	0.624 (0.366–1.065)	0.084

In the second analysis, lipid parameters were transformed from continuous variables into clinically defined categorical variables using common clinical thresholds, as detailed in Supplementary Table 1. This analysis was performed to determine whether abnormal lipid status itself was associated with the outcome. The results are shown in Table 5. Reduced HDL-C, elevated TG, and elevated LDL-C were all associated with increased odds of the outcome. After further inclusion of metabolic factors such as hyperglycemia and elevated uric acid, these associations remained relatively stable. These findings indicate that the associations observed in the main analysis were not entirely dependent on continuous-variable modeling. When classification criteria closer to clinical interpretation were used, abnormal lipid status remained statistically associated with diagnoses related to sensorineural hearing loss.

**Table 5:** Sensitivity analysis using categorical lipid variables.

Model	Variable	OR (95%CI)	*p-*value
Categorical lipid metabolism model	Reduced HDL-C	2.524 (1.426–4.468)	0.001
Categorical lipid metabolism model	Elevated TG	1.708 (1.182–2.467)	0.004
Categorical lipid metabolism model	Elevated LDL-C	1.542 (1.065–2.231)	0.022
Categorical extended metabolic model	Reduced HDL-C	2.523 (1.421–4.477)	0.002
Categorical extended metabolic model	Elevated TG	1.612 (1.107–2.348)	0.013
Categorical extended metabolic model	Elevated LDL-C	1.531 (1.056–2.220)	0.025

### 3.5 Model fit and collinearity

To evaluate model fit and collinearity, Akaike information criterion (AIC), Bayesian information criterion (BIC), McFadden’s pseudo-*R*^2^, likelihood-ratio tests, and maximum variance inflation factors (VIFs) were examined. For direct comparison, the three main models were refitted in the same complete-case sample (*N* = 577). Compared with the basic demographic model, the addition of TG, HDL-C, and LDL-C reduced the AIC from 799.094 to 779.084 and significantly improved model fit (nested likelihood-ratio test, *p* < 0.001). Further inclusion of BG, UA, and DBP did not provide significant additional improvement (*p* = 0.522). Maximum VIF values remained below 2, indicating no obvious multicollinearity. Detailed model-fit indices are presented in Supplementary Table 2.

### 3.6 Nonlinear and dose–response analyses

In the multivariable logistic regression models, TG, HDL-C, and LDL-C were included as continuous variables, primarily reflecting the average change in the odds of the outcome for each 1 mmol/L increase in lipid level. However, this approach assumes an approximately linear relationship between the exposure and the outcome on the logit scale, and may not fully capture changes in the effect across low or high concentration ranges. To further characterize the shape of the relationship between lipid levels and the outcome, restricted cubic spline analysis and quartile-based trend analysis were performed for the main lipid parameters.

Restricted cubic spline analysis was conducted using four knots located at the 5th, 35th, 65th, and 95th percentiles. The median value of each lipid parameter was used as the reference, and the models were adjusted for age, sex, and BMI. The results are presented in Table 6 and Figure 2. The RCS analysis showed overall associations of TG and HDL-C with the outcome. For TG, the test for nonlinearity did not reach statistical significance, suggesting that its relationship with the outcome was closer to a linear positive association. For HDL-C, both the overall association and the test for nonlinearity reached statistical significance, suggesting a possible nonlinear negative relationship between HDL-C and the outcome; that is, the increase in the odds of the outcome was more pronounced when HDL-C levels were lower. For LDL-C, neither the overall association nor the test for nonlinearity reached statistical significance in the RCS analysis, although the overall direction of the curve was generally consistent with the results of the multivariable logistic regression models.

**Table 6:** Nonlinear associations and quartile-based trend analyses of major lipid parameters.

Variable	Overall *p* for RCS	*p* for nonlinearity	Trend OR (95% CI)	*p* for trend	Overall association *p-*value
TG	0.001	0.373	1.340 (1.145–1.569)	<0.001	0.003
HDL-C	<0.001	0.032	0.720 (0.608–0.851)	<0.001	0.001
LDL-C	0.101	0.357	1.126 (0.970–1.307)	0.118	0.334

An overall *p*-value for RCS <0.05 indicates a statistically significant association. A *p*-value for nonlinearity <0.05 indicates evidence of a nonlinear relationship. A *p*-value for trend <0.05 was considered statistically significant.

**Figure 2:**
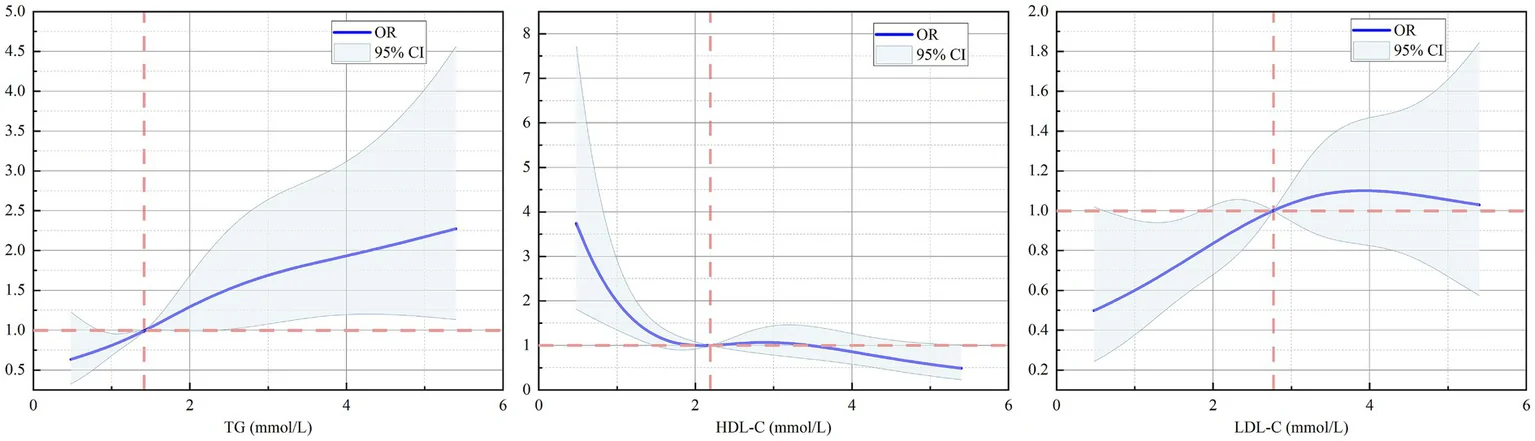
Restricted cubic spline curves for major lipid parameters and the outcome.

Quartile-based trend analysis further supported these findings. As shown in Figure 3, increasing quartiles of TG were associated with progressively higher odds of the outcome, whereas increasing quartiles of HDL-C were associated with lower odds of the outcome. The trend analysis for LDL-C did not reach statistical significance. Taken together, the RCS and quartile-based analyses suggested relatively more consistent dose–response evidence for TG and HDL-C. Although LDL-C was statistically significant in the multivariable logistic regression models, the dose–response evidence for LDL-C was relatively weak and requires further validation in studies with larger sample sizes.

**Figure 3:**
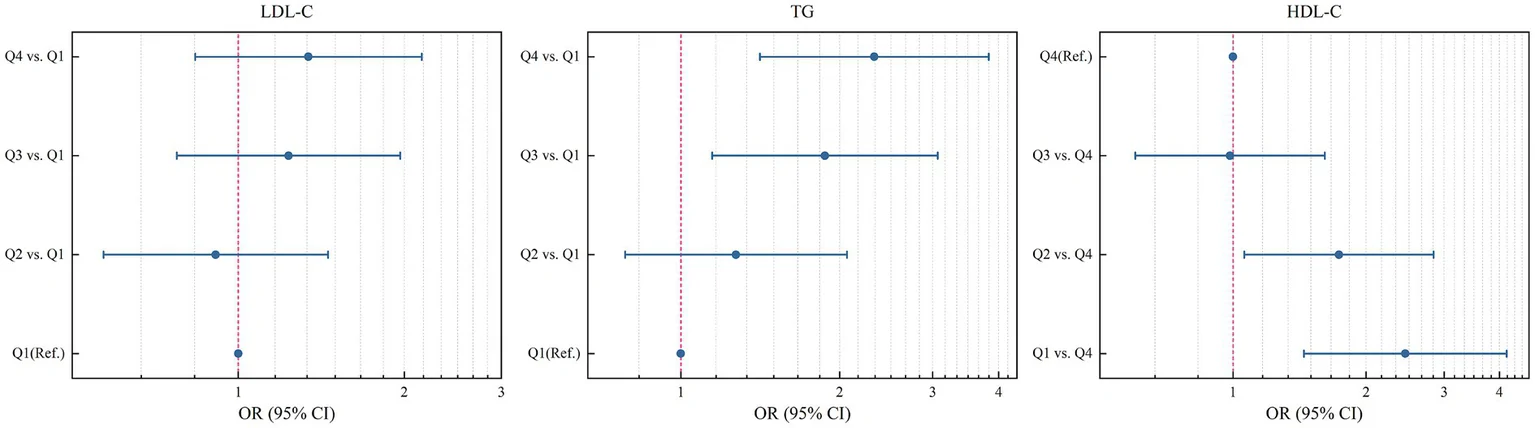
Quartile plot of lipid parameters.

### 3.7 Subgroup and interaction analyses

To explore whether the associations between the main lipid parameters and the outcome differed substantially across different populations, subgroup analyses were performed according to sex, BMI, and age. Interaction tests were further conducted to determine whether these factors acted as effect modifiers. The results are shown in Figure 4.

**Figure 4:**
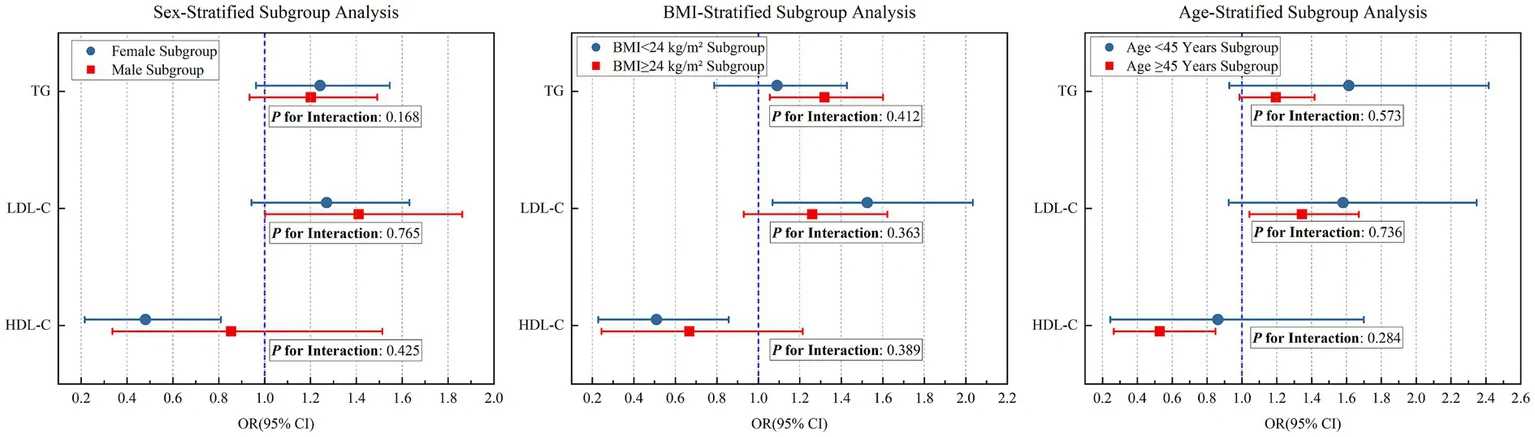
Multivariable associations of major lipid parameters across subgroups.

The directions of association for TG, LDL-C, and HDL-C were generally consistent across sex, BMI, and age strata. Although some subgroup-specific estimates reached statistical significance, none of the interaction tests were significant (Supplementary Table 3). These findings were therefore considered exploratory.

## 4 Discussion

In this retrospective case–control study of 580 otologic inpatients, the case group had higher TG, LDL-C, BMI, and UA levels and lower HDL-C levels than the control group. After multivariable adjustment, higher TG and LDL-C levels were associated with higher odds of SNHL-related diagnoses, whereas HDL-C showed an inverse association.

The pathological basis of SNHL mainly involves cochlear hair cells, the auditory nerve, or the central auditory pathway. Its pathogenesis is complex and may be associated with inner-ear microcirculatory disturbance, oxidative stress, inflammatory responses, immune abnormalities, and metabolic disorders (3–5). The blood supply of the inner ear has the characteristics of an end-arterial system, with relatively limited collateral circulation; accordingly, the cochlea is highly vulnerable to ischemia, hypoxia, increased blood viscosity, and fluctuations in the local metabolic environment (4–6, 11). Systemic abnormalities in lipid metabolism may therefore participate in the onset or progression of SNHL by affecting vascular endothelial function, microcirculatory perfusion, and inflammatory and oxidative stress status. These mechanisms provide biological plausibility for the observed associations, although the present study cannot establish a causal pathway.

Regarding specific lipid parameters, elevated TG may reflect abnormalities in triglyceride-rich lipoproteins and their remnant metabolism. These lipoprotein remnants may participate in chronic low-grade inflammation, endothelial injury, and atherosclerosis, and may adversely affect cochlear microcirculation by increasing blood viscosity, impairing microvascular perfusion, and aggravating local metabolic stress (12, 13). Elevated LDL-C is closely related to lipid deposition, oxidative modification, and vascular endothelial injury, and is an important factor in atherosclerotic vascular damage (14). Given the anatomical background of terminal blood supply and limited collateral circulation in the inner ear, elevated TG and LDL-C may jointly compromise the stability of inner-ear perfusion, providing a plausible biological explanation for their positive associations with SNHL-related diagnoses in the present study.

In contrast to TG and LDL-C, HDL-C showed a negative association in this study. In addition to its role in reverse cholesterol transport, high-density lipoprotein (HDL) has anti-inflammatory and antioxidant properties, improves endothelial function, and helps maintain vascular homeostasis (15). Reduced HDL-C may therefore indicate not only an abnormal lipid profile but also diminished vascular protective capacity and antioxidant reserve. For cochlear structures that are particularly sensitive to ischemia, hypoxia, and oxidative stress, reduced HDL-C may render the inner-ear microenvironment more susceptible to metabolic disturbance and vascular dysfunction. Across multivariable, categorical, RCS, and quartile analyses, HDL-C showed a broadly consistent inverse relationship with SNHL-related diagnoses, although causal interpretation is not warranted.

Previous studies have reported potential links between cardiovascular risk factors, metabolic syndrome, and sudden sensorineural hearing loss (16). Systematic reviews and meta-analyses have shown that patients with SNHL often have a higher burden of cardiovascular risk factors, including hypertension, abnormal glucose metabolism, and lipid metabolic abnormalities (17, 18); some studies have also suggested that the risks of stroke and myocardial infarction may be increased after SNHL (19). Lam et al. (20) likewise suggested that the occurrence and recovery of SNHL may be adversely affected in patients with metabolic syndrome. These findings support the view that SNHL may not be confined to local otologic pathology, but may also be related to systemic vascular risk and metabolic status. In the present study, contemporaneous otologic inpatients were used as controls, and similar directions of association were observed in this otologic inpatient control setting, providing additional real-world evidence on metabolic characteristics in otorhinolaryngology inpatients.

The present study further evaluated the robustness and interpretative scope of the findings through several supplementary analyses. When the outcome was restricted to sudden deafness or sudden hearing loss, TG and LDL-C remained positively associated with the outcome, whereas HDL-C retained an inverse association but no longer reached statistical significance. The preservation of the association directions suggests that the main findings were not solely dependent on the broader outcome definition, while the reduced number of cases likely contributed to greater uncertainty in the effect estimates. Similar directions of association were also observed when the control group was restricted to patients with external- or middle-ear disorders, although the confidence intervals widened and statistical significance was not retained, which may partly reflect the smaller comparator sample. In addition, when lipid parameters were analyzed using clinically defined categories, elevated TG, elevated LDL-C, and reduced HDL-C remained associated with the outcome, suggesting that the main findings were not dependent solely on continuous-variable modeling. RCS and quartile-based analyses provided relatively consistent dose–response evidence for TG and HDL-C, whereas the corresponding evidence for LDL-C was weaker despite its significant association in the multivariable models. Finally, subgroup analyses showed generally consistent directions of association across sex, age, and BMI strata, and no statistically significant interactions were identified. Taken together, these supplementary analyses generally supported the robustness of the main findings, while also indicating greater uncertainty for some estimates under more restrictive analytical conditions.

This study also has clinical implications. Previous guidelines and reviews have indicated that the diagnosis and management of SNHL require early audiological evaluation, together with comprehensive assessment of disease course, accompanying symptoms, and relevant risk factors (21, 22). Prognosis is influenced by the initial hearing threshold, timing of treatment, and multiple clinical factors; therefore, no single metabolic parameter can fully explain its occurrence or prognosis (23). Lipid and related metabolic profiles may provide additional information for characterizing the metabolic background of patients with SNHL-related diagnoses. Elevated TG and LDL-C and reduced HDL-C may indicate a more pronounced background of metabolic abnormality.

This study has several limitations. First, as a single-center retrospective study, residual confounding cannot be excluded. Fasting blood glucose was included in the adjusted model, whereas diagnosed diabetes mellitus, smoking status, cardiovascular disease, and pre-admission lipid-lowering medication use were not systematically available as structured variables. Second, lipid parameters were based on a single fasting measurement during hospitalization. The exact timing of blood sampling relative to corticosteroid or other acute treatment could not be consistently reconstructed, and a single measurement may not fully represent long-term lipid status. Third, the control group consisted of heterogeneous otologic inpatients rather than healthy controls. Some control conditions, such as tinnitus and vestibular disorders, may themselves be associated with vascular or metabolic abnormalities. Fourth, detailed audiological parameters were not uniformly available in the analytical dataset; therefore, the findings should primarily be interpreted at the level of clinically assigned SNHL-related diagnoses.

## 5 Conclusion

This study showed that, among otologic inpatients in the Department of Otorhinolaryngology, higher TG and LDL-C levels and lower HDL-C levels were associated with SNHL-related diagnoses after multivariable adjustment. These findings were generally supported by supplementary analyses, although causal inference is not possible. Prospective studies are needed to further clarify these associations across specific SNHL subtypes.

## Data Availability

The original contributions presented in the study are included in the article/Supplementary material, further inquiries can be directed to the corresponding author.
