# Supplementary material for: Association between dyslipidemia and diagnoses related to sensorineural hearing loss: a retrospective case–control study
Source: Front Neurol. 2026 Oct 28;17:1979454. doi: 10.3389/fneur.2026.1979454 (PMC13751420; doi:10.3389/fneur.2026.1979454)
Supplement: Supplementary file 1 [file Data_Sheet_2.docx]

**Supplementary File 2. Codebook for Categorical Variables**

**This codebook accompanies Supplementary File 1, “De-identified Raw Data”.** It provides the factor labels and definitions for categorical variables, especially those recorded numerically in the raw dataset. Numeric codes should be interpreted according to the factor labels below.

**Dataset:** 580 de-identified otologic inpatient records. **Note:** Continuous variables such as age, blood lipid measures, BMI, blood pressure, blood glucose, creatinine, uric acid, and urea are retained as continuous numeric variables in the raw data.

| **Variable name in raw data** | **Variable label** | **Coding / factor labels** | **Definition / cutoff used** |
| --- | --- | --- | --- |
| Sex | Sex | Female = female; Male = male | Text-coded categorical variable. |
| Outcome variable | Case-control outcome | 0 = control; 1 = case | Cases were patients with diagnoses related to sudden deafness, sudden hearing loss, sensorineural hearing loss, or high-frequency hearing loss. Controls were otologic inpatients without these outcome diagnoses. |
| Older age group | Age group | 0 = younger age group; 1 = older age group | The cutoff used in the analysis was 45 years: 0 = <45 years; 1 = ≥45 years. |
| Overweight status | Overweight status | 0 = no; 1 = yes | Overweight was defined as BMI ≥24 kg/m². |
| Hypertriglyceridemia status | Hypertriglyceridemia | 0 = no; 1 = yes | Hypertriglyceridemia was defined as triglycerides (TG) ≥1.70 mmol/L. |
| Elevated total cholesterol status | Elevated total cholesterol | 0 = no; 1 = yes | Elevated total cholesterol was defined as total cholesterol (TC) ≥5.20 mmol/L. |
| Low HDL-C status | Low high-density lipoprotein cholesterol | 0 = no; 1 = yes | Low HDL-C was defined as high-density lipoprotein cholesterol (HDL-C) <1.00 mmol/L. |
| Elevated LDL-C status | Elevated low-density lipoprotein cholesterol | 0 = no; 1 = yes | Elevated LDL-C was defined as low-density lipoprotein cholesterol (LDL-C) ≥3.40 mmol/L. |
| Hyperglycemia status | Hyperglycemia | 0 = no; 1 = yes | Hyperglycemia was defined as blood glucose (BG) ≥6.10 mmol/L. |
| Hypertension status | Hypertension | 0 = no; 1 = yes | Hypertension status was coded as yes when systolic blood pressure (SBP) was ≥140 mmHg and/or diastolic blood pressure (DBP) was ≥90 mmHg. |
| Elevated creatinine status | Elevated creatinine | 0 = no; 1 = yes | Elevated creatinine was defined as creatinine (CRE) >104 µmol/L in males and >84 µmol/L in females. |
| Elevated uric acid status | Elevated uric acid | 0 = no; 1 = yes | Elevated uric acid was defined as uric acid (UA) ≥360 µmol/L. |
| Elevated urea status | Elevated urea | 0 = no; 1 = yes | Elevated urea was defined as urea ≥7.10 mmol/L. |

**Abbreviations:** TG, triglycerides; TC, total cholesterol; HDL-C, high-density lipoprotein cholesterol; LDL-C, low-density lipoprotein cholesterol; BMI, body mass index; SBP, systolic blood pressure; DBP, diastolic blood pressure; BG, blood glucose; CRE, creatinine; UA, uric acid.
