# Supplementary material for: Association between dyslipidemia and diagnoses related to sensorineural hearing loss: a retrospective case–control study
Source: Front Neurol. 2026 Oct 28;17:1979454. doi: 10.3389/fneur.2026.1979454 (PMC13751420; doi:10.3389/fneur.2026.1979454)
Supplement: Supplementary file 2 [file Supplementary_file_1.docx]

**Supplementary Materials Table**

**Supplementary Table 1 Thresholds Used to Convert Continuous Variables Into Categorical Variables**

| **Variable** | **Categorical Variable** | **Threshold** | **Coding** |
| --- | --- | --- | --- |
| TG | Elevated TG | TG ≥ 1.7 mmol/L | 1 |
| TC | Elevated TC | TC ≥ 5.2 mmol/L | 1 |
| LDL-C | Elevated LDL-C | LDL-C ≥ 3.4 mmol/L | 1 |
| HDL-C | Reduced HDL-C | HDL-C ≤ 1.0 mmol/L | 1 |
| BMI | Elevated BMI | BMI ≥ 24 kg/m² | 1 |
| BG | Elevated BG | Fasting blood glucose ≥ 6.1 mmol/L | 1 |
| SBP/DBP | Elevated blood pressure | SBP ≥ 140 mmHg or DBP ≥ 90 mmHg | 1 |
| UA | Elevated UA | UA > 420 | 1 |
| CRE | Elevated CRE | >104 in men and >84 in women | 1 |

**Supplementary Table 2 Model Fit and Collinearity Diagnostics for Logistic Regression Models**

| Model | N | Outcome = 1 | AIC | BIC | McFadden’s pseudo-R² | LLR *P* Value | Maximum VIF |
| --- | --- | --- | --- | --- | --- | --- | --- |
| Basic demographic model | 580 | 284 | 802.947 | 820.399 | 0.011 | 0.031 | 1.042(male sex) |
| Lipid metabolism model | 577 | 283 | 779.084 | 809.589 | 0.043 | <0.001 | 1.500(HDL-C) |
| Extended metabolic model | 577 | 283 | 782.835 | 826.413 | 0.046 | <0.001 | 1.542(HDL-C) |
| Sudden-deafness-only lipid metabolism model | 524 | 231 | 711.496 | 741.326 | 0.030 | 0.001 | 1.511(HDL-C) |
| Sudden-deafness-only extended metabolic model | 524 | 231 | 714.977 | 753.330 | 0.031 | 0.005 | 1.549(HDL-C) |
| Categorical lipid metabolism model | 577 | 283 | 774.849 | 805.354 | 0.049 | <0.001 | 1.166(reduced HDL-C = 1) |
| Categorical extended lipid model | 577 | 283 | 774.365 | 813.586 | 0.054 | <0.001 | 1.271(elevated UA = 1) |

Note: Lower AIC and BIC values indicate relatively better model fit. Higher McFadden’s pseudo-R² values indicate greater improvement in model fit relative to the null model. LLR *P* < 0.05 indicates that the overall model is statistically significant.

**Supplementary Table 3** **Subgroup and Interaction Analyses**

| Variable | Subgroup | OR (95% CI) | *P* Value | *P* for Interaction |
| --- | --- | --- | --- | --- |
| TG | Men | 1.180 (0.933-1.491) | 0.167 | 0.765 |
| TG | Women | 1.219 (0.962-1.545) | 0.101 | 0.765 |
| TG | BMI ≥24 kg/m² | 1.299 (1.055-1.601) | 0.014 | 0.412 |
| TG | BMI <24 kg/m² | 1.059 (0.787-1.426) | 0.704 | 0.412 |
| TG | Age ≥45 years | 1.181 (0.985-1.416) | 0.072 | 0.573 |
| TG | Age <45 years | 1.497 (0.927-2.417) | 0.099 | 0.573 |
| LDL-C | Men | 1.366 (1.002-1.862) | 0.048 | 0.168 |
| LDL-C | Women | 1.239 (0.942-1.631) | 0.125 | 0.168 |
| LDL-C | BMI ≥24 kg/m² | 1.228 (0.929-1.622) | 0.149 | 0.363 |
| LDL-C | BMI <24 kg/m² | 1.474 (1.068-2.034) | 0.018 | 0.363 |
| LDL-C | Age ≥45 years | 1.320 (1.043-1.670) | 0.021 | 0.736 |
| LDL-C | Age <45 years | 1.472 (0.924-2.346) | 0.104 | 0.736 |
| HDL-C | Men | 0.712 (0.336-1.513) | 0.378 | 0.425 |
| HDL-C | Women | 0.417 (0.215-0.809) | 0.010 | 0.425 |
| HDL-C | BMI ≥24 kg/m² | 0.544 (0.244-1.214) | 0.137 | 0.389 |
| HDL-C | BMI <24 kg/m² | 0.442 (0.228-0.857) | 0.016 | 0.389 |
| HDL-C | Age ≥45 years | 0.473 (0.264-0.848) | 0.012 | 0.284 |
| HDL-C | Age <45 years | 0.644 (0.244-1.699) | 0.374 | 0.284 |

Note: Age was stratified at 45 years. BMI was stratified according to the overweight variable in the dataset, corresponding to BMI ≥24 kg/m² and BMI <24 kg/m². The *P* value for interaction was derived from a joint likelihood ratio test. ORs, 95% CIs, and subgroup-specific *P* values were obtained from multivariable logistic regression models within each subgroup and were used to describe the direction and strength of association for the main lipid parameters across different subgroups. *P* for interaction was derived from full-sample logistic regression models including product terms between lipid parameters and stratification variables, and was used to evaluate differences in effects between subgroups. Interaction-term ORs were not interpreted as subgroup effect estimates; therefore, only *P* values for interaction are reported. For continuous TG, LDL-C, and HDL-C, ORs indicate the change in the odds of the outcome per 1 mmol/L increase in the corresponding parameter. *P* < 0.05 was considered statistically significant.
